# The effect of the delta SARS-CoV-2 variant on maternal infection and pregnancy

**DOI:** 10.1016/j.isci.2022.104295

**Published:** 2022-04-25

**Authors:** Athina Samara, Asma Khalil, Patrick O’Brien, Eric Herlenius

**Affiliations:** 1 Department of Women’s and Children’s Health, Karolinska Institutet, Stockholm, Sweden; 2 Astrid Lindgren Children’s Hospital, Karolinska University Hospital, Stockholm, Sweden; 3 Fetal Medicine Unit, St George’s Hospital, St George’s University of London, London, UK; 4 Vascular Biology Research Centre, Molecular and Clinical Sciences Research Institute, St George’s University of London, London, UK; 5 Fetal Medicine Unit, Liverpool Women’s Hospital, University of Liverpool, Liverpool, UK; 6 The Royal College of Obstetricians and Gynaecologists, London, UK; 7 University College London Hospitals NHS Foundation Trust, London, UK

**Keywords:** Virology, Pregnancy

## Abstract

A greater proportion of pregnant women with COVID-19 have mild disease compared with their non-pregnant counterparts. Paradoxically, however, they are at higher risk of developing severe disease, requiring respiratory support and admission to intensive care. The delta SARS-Cov-2 variant is associated with increased risk of hospitalization and morbidity in unvaccinated pregnant populations. However, it is not known whether the worse pregnancy outcomes associated with the delta variant are due to a direct effect of the virus on the pregnancy, or whether this effect is mediated through more severe maternal infection. Here, we synthesize studies of COVID-19 pregnancies, focusing on the different routes of SARS-CoV-2 infection of lung and placenta, and the mechanisms of syncytial formation for each SARS-CoV-2 variant. To delineate COVID-19 complications in pregnant women, future studies should explore whether the delta variant causes greater placental infection compared to other variants and contributes to increased syncytial formation.

## Introduction

Although pregnant women with COVID-19 usually display mild to moderate symptoms when compared to non-pregnant women with the same risk profile, they are at higher risk of developing severe disease, requiring respiratory support and admission to intensive care (ICU) (Knight et al., 2021; Villar et al., 2021; Allotey et al., 2020; Engjom et al., 2022; Donati et al., 2022; Overtoom et al., 2021). The USA Centers for Disease Control and Prevention (CDC) reported an apparent increase in the ratio of COVID-19-associated deaths per 1,000 cases among pregnant women as the delta variant became predominant (Kasehagen et al., 2021). That amounted to 5 vs 25 deaths per 1,000 SARS-CoV-2 infections during pregnancy, in the pre-delta (Mar 2020–Jun 2021) compared to the delta-predominant period (Jul–Oct 2021). Several studies reported that, compared with the other widespread variants, the delta variant is associated with increased risk of hospitalization and morbidity in unvaccinated pregnant populations (Vousden et al., 2021). The CDC reported (DeSisto et al., 2021) that the risk of stillbirth was 2-fold higher in pregnant women with COVID-19 compared with those without the infection.

What is not known, however, is whether the worse pregnancy outcomes associated with the delta variant are due to a direct effect of the virus on the pregnancy, or whether this effect is mediated through more severe maternal infection. The delta variant has been associated with greater viral loads than the alpha variant (Luo et al., 2021), but the pathophysiological mechanisms affecting pregnancy outcomes remain unclear.

## Epidemiological studies reporting an association between the delta variant and worse maternal infection and pregnancy outcomes

A national UK prospective cohort study showed that, after adjusting for pre-existing medical conditions and sociodemographic variables, the proportion of symptomatic pregnant women hospitalized with moderate to severe COVID-19 increased significantly from the wild type (24%) to alpha (36%) to the delta period (45%) (Vousden et al., 2021). Furthermore, pregnant women admitted during the delta wave had increased risk (compared to those hospitalized during the alpha wave) of having pneumonia and had non-significant increases in the need for respiratory support and ICU admission (Vousden et al., 2021) and death (UKOSS MBRRACE Infographic v13, 2021). A US study also found that the proportion of severe to critical disease resulting in ICU admission was greater in the delta cohort compared to the pre-delta cohort (Seasely et al., 2021). Moreover, in this study, the rates of adverse pregnancy outcomes including cesarean delivery, preterm birth, and neonatal ICU admission were also higher in the delta cohort. Increased morbidity was also observed in pregnancy with COVID-19 during the Delta surge (Adhikari et al., 2022a).

A meta-analysis evaluated the severity of disease caused by the SARS-COV-2 variants of concern (VOCs) in the general population from June 1, 2020 to October 15, 2021. This showed that the beta and delta variants pose a greater risk of hospitalization, ICU admission, and mortality compared to the alpha, gamma, and wild-type variants (Lin et al., 2021). Findings from the UK report suggested that during the periods of alpha and delta variant dominance, COVID-19 was associated with more severe maternal infection and worse pregnancy outcomes compared to the period of wild-type dominance (Vousden et al., 2022). Moreover, recent US reports showed that the delta and omicron variants were associated with increased SARS-CoV-2 infections in pregnancy (Adhikari et al., 2022a; 2022b). Among those, the majority occurred in unvaccinated pregnant women and, after adjusting for prior vaccination, the predominance of the delta variant was associated with increased, and omicron with decreased, severity of illness (Adhikari et al., 2022a; 2022b). The finding that most women admitted with SARS-CoV-2-related symptoms were unvaccinated was also reported (Vousden et al., 2022; Birol Ilter et al., 2022).

## Transplacental transfer of neutralizing antibodies against both wild type and delta variants in vaccinated women

Full vaccination during pregnancy is essential but data are limited regarding transplacental antibody transfer after vaccination with the currently available vaccines. A small study (n = 29) evaluating maternal and umbilical cord blood on the day of birth assessed neutralizing antibody levels for both the wild type and delta variant and showed a pronounced reduction for the delta variant (Shen et al., 2022).

## Severity of maternal COVID-19 does not consistently correlate with placental SARS-CoV-2 viral load

Although vertical transmission of SARS-CoV-2 is rare, has been observed (Zaigham and Andersson, 2020; Kasehagen et al., 2021) and placental pathology might be present even in late term asymptomatic cases (Jaiswal et al., 2021). Several reports of severe placental SARS-CoV-2 infection describe malperfusion and diffuse inflammatory histological changes, including massive perivillous fibrin depositions, necrosis of syncytiotrophoblast, and diffuse chronic intervillositis (Mao et al., 2022; Schwartz et al., 2021; Schwartz and Morotti, 2020). While these severe placental histopathological findings are not unique to maternal COVID-19 (Husen et al., 2021), the severity of maternal COVID-19 has been correlated with placental SARS-CoV-2 viral load (Rangchaikul and Venketaraman, 2021). A histological analysis comparing placentas of 28 uninfected and 85 women with symptomatic SARS-CoV-2 infection in pregnancy showed severe vascular remodeling of the placental arteries, thickened placental vascular walls, and narrowed lumen (Gychka et al., 2021). The vascular remodeling was associated with increased smooth muscle cell proliferation and fibrosis.

Pregnant women with gestational diabetes mellitus are nine times as likely as those without diabetes to be infected with the delta variant and are three times more susceptible than those with cardiovascular disease or hypertension (Mamun and Khan, 2021). Women with gestational diabetes are also more vulnerable to infection with the delta variant than with wild type or alpha variants (Mamun and Khan, 2021). SARS-CoV-2 was more commonly found in placentas of COVID-19-positive mothers with hypertensive disorders of pregnancy (HDP) compared to those without HDP (Fabre et al., 2021). The authors of this study also suggest the possibility that SARS-CoV-2 infection during pregnancy could trigger HDP through persistent placental infection causing placental damage (Fabre et al., 2021), but did not offer a mechanistic explanation.

In a review, Rangchaikul and Venketaraman suggested some parameters that may predispose the pregnant patient to greater susceptibility to and severity of SARS-CoV-2. These include, among others, hampering of cell-mediated immune clearance of SARS-CoV-2, altered immunomodulation by progesterone, coagulation, and complement-associated hyperinflammation (Rangchaikul and Venketaraman, 2021).

A recent report described two cases of intra-uterine fetal demise and a case of severe fetal distress after infection of unvaccinated mothers with the delta variant and mild COVID-19 disease severity (Shook et al., 2022). All mothers had viremia and high nasopharyngeal viral load, but also evidence of placental infection with the delta variant and features of SARS-CoV-2-induced placentitis. Similarly, another case report of third trimester fetal demise following infection with the delta variant, described an unvaccinated mother with mild COVID-19 symptoms, but the placental analysis showed intervillous inflammation, degeneration, loss of syncytiotrophoblastic layers, and syncytial knots (Guan et al., 2022).

## Formation of syncytia as a pathological feature of COVID-19

Cell-to-cell fusion that leads to the formation of a syncytium is a physiological mechanism that occurs in various cell types, such as myocytes. The same mechanism allows viruses to infect neighboring cells without the exocytosis of free virus, and virus-infected syncytia increase tissue damage. Infectious syncytia are formed by the attachment of virions to cells or cell-to-cell fusion and are typical of coronaviruses (Li et al., 2003; Buchrieser et al., 2020). Formation of syncytia is a central pathological feature of COVID-19. COVID-19 severity also correlates closely with lung damage, and syncytia are often observed in the lungs of patients who have developed fatal pneumonia (Sanders et al., 2021; Braga et al., 2021; Bussani et al., 2020). It has also been demonstrated that SARS-CoV-2-infected syncytia may affect cardiomyocytes (Bailey et al., 2021).

The rate of formation of syncytia by SARS-CoV-2 has been shown to be much faster than by SARS-CoV-1 (Mehta et al., 2020). Of note, this syncytium formation determines both the degree of virulence and induction of the cytokine storm by SARS-CoV-2 (Matsuyama et al., 2020; Xia et al., 2020), as seen with other viruses.

Regarding the size of the syncytia, it has been reported that the alpha and beta variants produced larger syncytia than both the original Wuhan strain and the D614G variant (one of the earliest variants associated with an increased rate of transmission) (Rajah et al., 2021). Another study compared the replication rate and cell-to-cell transmission infection pattern of SARS-CoV-2 variants *in vitro*. Of the cells positive for any variant, at least 43% were distributed as clusters of 2 or more cells, and the delta variant proved to have the highest percentage (>78%) of infected cells organized in clusters, relative to alpha (ca. 59%) or beta (ca. 69%) variants (Al-Beltagi et al., 2021).

**Figure 1:**
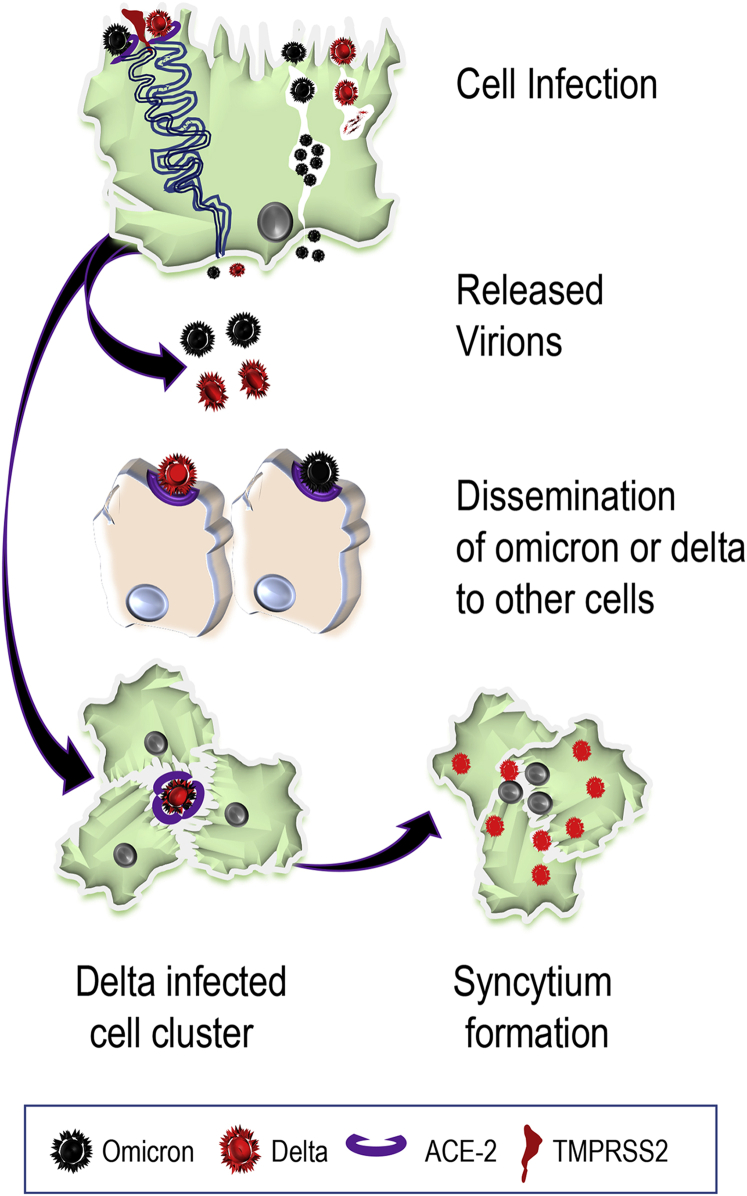
Delta SARS-CoV-2 variant efficiently enters cells via binding to the ACE2 receptor and activating cell membrane fusion using the host cell-surface protease TMPRSS2 This specific mechanism of infection of cells bearing both the ACE2 and TMPRSS2 (double ACE2+ TMPRSS2+ cell infection) allows the delta virions to bypass the endosome and not be destroyed by the host cell. On the other hand, omicron may enter cells in both a TMPRSS2-dependent and independent manner, as it can avoid the endosome, and may also infect ACE2 cells. Omicron enters cells through endocytosis, as its spike proteins drive the fusion of viral and endosomal membranes to facilitate insertion of the viral genome into the cytoplasm. Once the cell is infected by omicron, the virions that are replicated inside are finally released to disseminate other cells. In the case of the delta variant, when the spike protein is expressed on the surface of infected cells, it may interact with the ACE2 receptors on neighboring cells and form syncytia.

There is evidence, therefore, that the delta variant spike protein may increase cell-to-cell fusion (Arora et al., 2021) when compared to the wild type, the alpha, and the omicron variant (Suzuki et al., 2022). Regarding omicron, preliminary animal model studies show that it causes less severe disease accompanied by lower viral load in both the lower and upper respiratory tract (Bentley et al., 2021). Preliminary studies on this variant show reduced ability to induce syncytia in tissue culture, and that it may instead use (Meng et al., 2022) endosomal fusion through cathepsins (Peacock et al., 2022; Willett et al., 2022) (Figure 1).

## SARS-CoV-2 effect on placentation, syncytialization, and gas exchange

Placentation is a complex multi-step process that enables placental perfusion, during which trophoblast cells adhere, invade, and remodel spiral arteries. Using a process called syncytialization, the cytotrophoblasts fuse with the overlying giant multinucleated syncytiotrophoblasts and form the outer layer of the placental microvilli (Burton and Fowden, 2015; Robbins and Bakardjiev, 2012) rendering the tissue impermeable and enabling mother–child immune tolerance (Alasadi et al., 2019). The cytotrophoblasts may regenerate the syncytiotrophoblast if damaged, but if the process is significantly compromised, pathological conditions such as preeclampsia and intra-uterine fetal growth restriction (FGR) might ensue (Huppertz and Kingdom, 2004; Pötgens et al., 2002; Sankar et al., 2012). Increased numbers of syncytial knots, as seen in the placenta under conditions of hypoxia, hyperoxia, or in the presence of reactive oxygen species (ROS) (Heazell et al., 2007), and in preeclampsia (Redline and Patterson, 1995), were also observed with COVID-19 (Singh et al., 2021; Gao et al., 2021). This was possibly due to maternal vascular malperfusion; the increase in numbers of syncytial knots was positively correlated with disease severity, as is the case with vascular endothelial growth factor (VEGF) expression (Shchegolev et al., 2021).

There may be small amounts of fibrin deposition in normal placentas, which is increased in HDP (Fox, 1967). Massive intervillous fibrin deposition has also been reported as a hallmark of COVID-19 pregnancies. As systemic SARS-CoV-2 infection leads to hypoxia and reduced utero-placental perfusion, it induces focal necrosis and extensive fibrin deposition. This increased amount of intravillous and perivillous fibrin might result from impaired fibrinolytic capacity of the compromised maternal endothelium or from immune cell activation with subsequent pro-coagulation signals. As fibrin deposition further compromises maternal-fetal gas exchange, further research is needed to elucidate the underlying cellular and molecular mechanisms.

## Routes of SARS-CoV-2 infection in lung vs. placental cells

For a cell to be permissive to SARS-CoV-2, it must express the angiotensin-converting enzyme 2 (ACE2) receptor and have protease activity—transmembrane serine protease 2 (TMPRSS2) (Matsuyama et al., 2010). After transmission, SARS-CoV-2 can replicate in the respiratory and gastrointestinal tracts, and cause disease ranging from asymptomatic to severe. The virus may then spread to other organs via the bloodstream. The syncytiotrophoblast, as the outer surface of the placental villi, is bathed in maternal blood, so placental infection may occur. In the case of COVID-19, electron microscopy has documented membrane-bound vesicles filled with virions in the syncytiotrophoblast, which in some cases is necrotic (Birkhead et al., 2021; Hosier et al., 2020).

Transcriptomic analyses have showed that only 3%–6% of lung airway epithelial cells, which are considered the primary route of SARS-CoV-2 lung infection, co-express *ACE2* and *TMPRSS2* (Ziegler et al., 2020). snRNA-seq from lungs of fatal COVID-19 cases further supports the low expression of *ACE2/TMPRSS2* (Melms et al., 2021). However, these findings could be explained by increased cell death in those cells expressing *ACE2/TMPRSS2* and facilitating virus entry.

Other studies have assessed the expression of *ACE2* and *TMPRSS2* in the different cell types of the placental villi, to evaluate which are permissive to the entry of SARS-CoV-2. One showed that trophoblasts, but not the other main villous cell types, express *ACE2* and *TMPRSS2*, and that these cells are capable of *ACE2* endocytosis (Ouyang et al., 2021). ACE2 and TMPRSS2 expression in the first trimester placenta has been also been reported (Weatherbee et al., 2020). Another study using scRNA-seq analysis found that *ACE2* expression was abundant throughout gestation. A subset of syncytiotrophoblast and extravillous trophoblast cells (amounting to 14% in first trimester placentas and 15% of second trimester placentas) co-expressed ACE2/TMPRSS2 (Ashary et al., 2020).

## Other mechanisms potentially contributing to the complications of COVID-19 in pregnant women

A study that offered a mechanistic correlation to severe disease showed that the expression levels of placental *ACE2* (but not *TMPRSS2* or *Furin*) and the protein levels of IFITM1 and IFITM3 were higher in women with severe COVID-19 (Mourad et al., 2021). More comprehensive results from scRNA-seq in first trimester pregnant women with and without COVID-19 showed that villous trophoblast cells express low levels of *ACE2* and *TMPRSS2*, but high levels of *DDP4* (the MERS-CoV entry mediator) and *CTSL*, which, according to the authors (Constantino et al., 2021), could be non-canonical cell-entry mediators for SARS-CoV-2. These authors also suggest that changes in expression of the genes *DAAM1* and *PAICS* that code for proteins predicted to interact with SARS-CoV-2 proteins during pregnancy might be of importance. *DPP4* was among the scRNA-seq datasets of healthy placentas, expressed in all the cell types of the first trimester placenta and also in extravillous trophoblast in the second trimester (Ashary et al., 2020). *CTSL* expression was independently shown to be promoted after SARS-CoV-2 infection (Zhao et al., 2021). scRNA-seq analysis comparing healthy controls and COVID-19 cases showed that *CTSL* expression was more abundant than ACE2 expression in placental trophoblast cells, and this transcription was increased in decidual stromal cells and antigen-presenting cells in COVID-19 pregnancies (Lu-Culligan et al., 2021). This highlights the interaction between SARS-CoV-2 and other placental proteins, suggesting that SARS-CoV-2 may utilize multiple mediators to infect the placenta.

In addition, the detachment of syncytial knots is a source of transcriptionally active soluble fms-like tyrosine kinase-1 (sFlt-1) syncytial aggregates in the maternal circulation. Syncytiotrophoblast-derived syncytial knots, which become multinucleate syncytial aggregates that express the antiangiogenic protein sFlt-1, have previously been detected in the lungs of pregnant women, and the number of syncytial aggregates in the maternal lungs was higher in women with preeclampsia (Buurma et al., 2013). This process of accelerated syncytial knot formation, shedding, and aggregation and delivery of microparticles might contribute to the maternal vascular injury seen in COVID-19 lung pathology. However, the immune cascade potentially triggered by viral syncytia has not been clarified, either in the placenta or in other tissues.

Infection by the SARS-CoV-2 delta variant in pregnant women might induce increased pathogenicity via epigenomic regulation or other non-canonical entry routes. Future research should focus on comparison of the alveolar and placental barriers, cell-to-cell communication, and the cellular and molecular partners involved.

Key to syncytialization is the hypomethylation (Matousková et al., 2006) and expression of the syncytin genes producing syncytin-1 and 2 that are known to derive from human endogenous retroviruses (Dupressoir et al., 2012). Altered expression of syncytin-1 and 2 (Liu et al., 2018) was also correlated with placental pathophysiology, including in preeclampsia (Knerr et al., 2002; Vargas et al., 2011). Further studies are needed to elucidate the epigenome landscape changes during gestation in the SARS-CoV-2-infected placenta and lungs.

Unlike the alveolo-capillary barrier in the lungs, the syncytiotrophoblast expresses little or no caveolin-1 (Mohanty et al., 2010) or caveolin-2 or 3 (Lyden et al., 2001). The role of caveolin-1 in signal transduction could be of importance as it can induce inflammatory cascades via nuclear factor kappa-light-chain-enhancer of activated B cells (NFkB) and leukocyte activation (Celik et al., 2020), and its downregulation was recently documented in immature alveolar endothelial cells in fatal COVID-19 snRNA-analysis (Melms et al., 2021).

Intercellular communication via gap junctions is also necessary for trophoblastic cell fusion and syncytiotrophoblast formation. The involvement of the gap junction protein connexin-43 was immunodetected at the intercellular boundaries between aggregated cells; the expression disappeared after cellular fusion (Frendo et al., 2003). There is scant information on the role of the pannexins in the development of syncytiotrophoblast, but Pannexin-1 (Panx-1) channel opening is known to accelerate viral entry, replication, cell-to-cell spread, and inflammation (Malik and Eugenin, 2019). The involvement of Panx-1 in lungs has been assessed in the context of patients with COVID-19 suffering from hyperinflammation (Swayne et al., 2020) and the Panx-1 channel was shown to open in response to SARS-CoV-2 (Luu et al., 2021).

**Figure 2:**
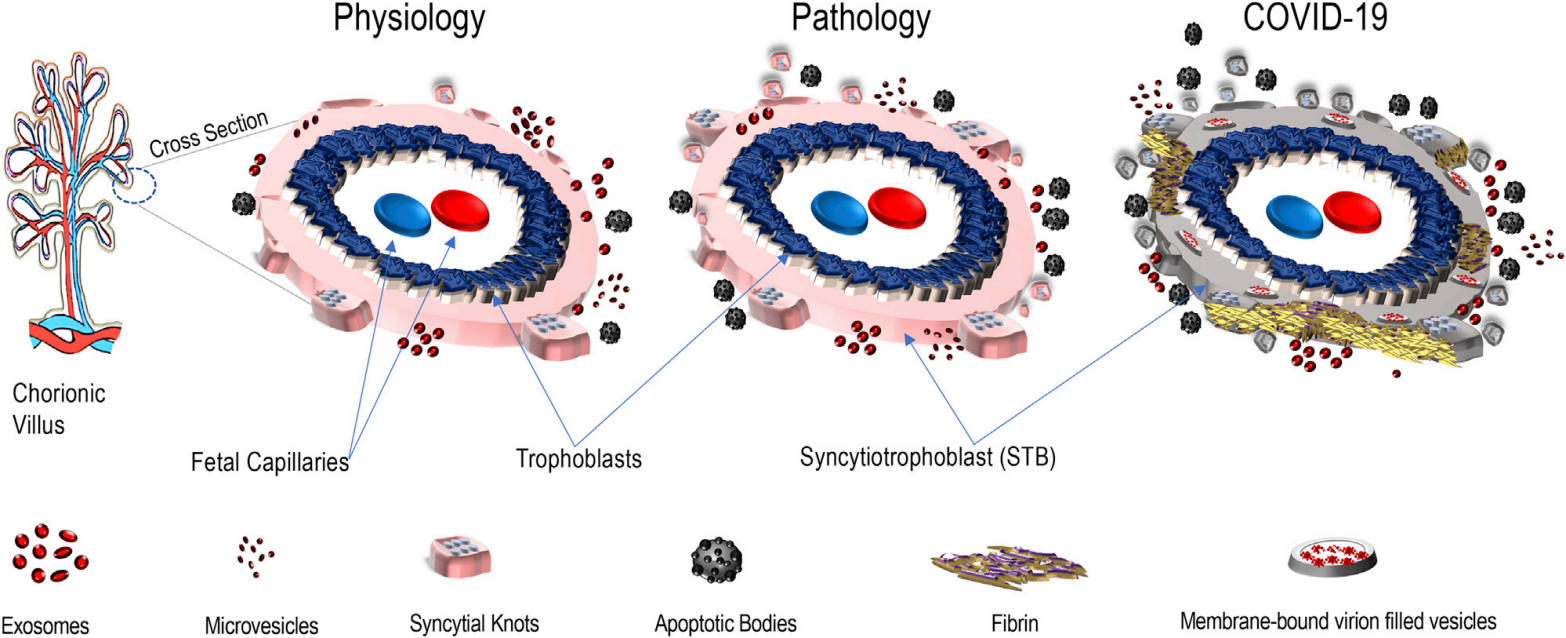
Cytotrophoblasts fuse with the overlying giant multinucleated syncytiotrophoblasts and form the outer layer of the placental microvilli to render the tissue impermeable and enable mother–child immune tolerance The cytotrophoblasts may regenerate the syncytiotrophoblast if damaged, but if the process is significantly compromised, pathological conditions such as preeclampsia and intra-uterine fetal growth restriction (FGR) might ensue. Increased numbers of syncytial knots, as seen in the placenta under conditions of hypoxia, hyperoxia, or in the presence of reactive oxygen species (ROS), and in preeclampsia, were also observed with COVID-19. There may be small amounts of fibrin deposition in normal placentas, which is increased in hypertensive disorders of pregnancy. But massive intervillous fibrin deposition has also been reported as a hallmark of COVID-19 pregnancies. As systemic SARS-CoV-2 infection leads to hypoxia and reduced utero-placental perfusion, it induces focal necrosis and extensive fibrin deposition. This increased amount of intravillous and perivillous fibrin might result from impaired fibrinolytic capacity of the compromised maternal endothelium or from immune cell activation with subsequent pro-coagulation signals. As fibrin deposition further compromises maternal-fetal gas exchange, further research is needed to elucidate the underlying cellular and molecular mechanisms.

Finally, the extensive syncytial formation due to SARS-CoV-2 infection might contribute to increased viral dissemination and cause greater and more extensive placental tissue damage (Figure 2). Moreover, the role of the increased numbers of circulating placental syncytia, their molecular cargo, and their effect and entrapment in lung vessels should be delineated. This would help further evaluate whether the ability of the delta variant to spread among cells, by direct contact via syncytia without the need for extracellular virion release, could explain how the virus partly escapes existing humoral response (Al-Beltagi et al., 2021). The suggested decreased fusogenicity of the omicron variant may also translate into more favorable pregnancy outcomes following infection (Meng et al., 2022; HKUMed 2022).

### Limitations of the study

The definitions of the severity of COVID-19 may vary among investigators. The data presented in this review are limited by the increasing rate of vaccination among pregnant women, the small number of published studies and preprints, and in some cases, the small sample size analyzed and the limited sequencing results in the studies of the pregnant patients.

## Outstanding questions

During the delta variant wave of the COVID-19 pandemic, the rate of admission of pregnant women to hospital and to ICU was increased compared to the first wave and the alpha wave of the pandemic. This could be due to various variant-specific factors, such as the increased delta variant transmissibility compared to the previous variants. Moreover, the significantly greater respiratory viral load of delta and its ability to form syncytia in the lungs could cause more severe disease and result in hypoxia. In addition, other patient-specific factors could be the lower vaccination rates in pregnant women compared to the general population, and pregnancy itself conferring greater susceptibility. Studies of changes in the epigenomic landscape and transcriptome, together with studies of placental histology, should address whether the delta variant causes greater placental infection and contributes to increased syncytia formation. This type of analysis would delineate whether the increased incidence of adverse pregnancy outcomes seen with the delta variant is due to more severe systemic maternal infection or due to a direct effect of this variant on the fetus or placenta.

## Search strategy and selection criteria

Data for this Review were identified by searches of Pubmed/Medline, Current Contents, BiorXiv, and references from relevant articles and preprints, using the search terms “pregnancy”, “COVID-19”, “delta variant”, “omicron”, and “syncytia”.
